# Synthesis and evaluation of polymeric micelle containing piperacillin/tazobactam for enhanced antibacterial activity

**DOI:** 10.1080/10717544.2019.1693708

**Published:** 2019-12-04

**Authors:** Milani Morteza, Salehi Roya, Hamishehkar Hamed, Zarebkohan Amir, Akbarzadeh Abolfazl

**Affiliations:** a Infectious and Tropical Diseases Research Center, Tabriz University of Medical Sciences, Tabriz, Iran;; b Drug Applied Research Center, Tabriz University of Medical Sciences, Tabriz, Iran;; c Department of Medical Nanotechnology, Faculty of Advanced Medical Sciences, Tabriz University of Medical Sciences, Tabriz, Iran

**Keywords:** *P. aeruginosa*, piperacillin/tazobactam, polymeric micelle, antibiotic resistance

## Abstract

Infections caused by multidrug-resistant bacteria such as *P. aeruginosa* are important therapeutic complications. Piperacillin/Tazobactam is considered a safe antimicrobial agent. But we should not ignore the prevalence of resistant strains to this drug. In this work, a new polymeric micelle composed of Piperacillin/Tazobactam-loaded Poly (ethylene glycol) methyl ether-block-poly (lactide-co-glycolide) (PLGA-PEG) was developed to improve the antimicrobial performance of P/T. The SEM and TEM studies of PLGA-PEG micelle showed, semi-spherical morphology with a mean diameter of below 30 nm. Zeta potential results indicated that the surface charge of PLGA-PEG micelle was −2.98 mV, while after encapsulation of P/T, the surface charge decreases to −4.13 mV. Clinical strains of *P. aeruginosa* were isolated and their resistance pattern against different antibiotics was evaluated. The MIC of free and P/T -Loaded PLGA-PEG micelles was determined. Also, the effect of free or P/T micelle against minimal biofilm eradication concentration and motility inhibition was evaluated. The bacterial isolates were resistant to most common antibiotics. The MIC of the free drug form and micelle form ranged from 4 to 512 µg/ml and 2 to 256 µg/ml, respectively. Generally, micelle showed more effective antibiofilm activities, inhibition of bacterial motility and reducing the MIC than that free drug form.

## Introduction

*Pseudomonas aeruginosa* is a Gram-negative, rod-shaped bacteria with the ability to survive in a variety of environmental conditions. In most cases, Pseudomonas infections are acquired from the hospital environment. Thus this microorganism is an important nosocomial pathogen (Fujii et al., 2014). Among infections caused by gram-negative bacilli, *P. aeruginosa* plays an important role especially in patients with severe illness and in immunocompromised patients. Therefore, control of infections caused by this bacterium is critical. Due to antibiotic resistance, physicians have encountered major challenges in the treatment of infections caused by gram-negative pathogens, especially Pseudomonas infections (Pena et al., 2009; El Zowalaty et al., 2015; Sligl et al., 2015).

Antibiotic treatment of this pathogen is extremely difficult due to multiple resistance mechanisms, such as b-lactamases, efflux pumps, and the impermeability of the outer membrane (Bassetti et al., 2018). In fact, this leads to a serious limitation of the options for the treatment of *P. aeruginosa* infections. Nowadays several antibiotics are used to treat *P. aeruginosa* infections. Piperacillin is a potent, broad-spectrum ureidopenicillin that is used against gram-negative, gram-positive and anaerobic bacteria. When combined with beta-lactamase inhibitors such as tazobactam, it demonstrates a broader spectrum of activity against lactamase-producing bacteria. For its spectrum of activity, Piperacillin/tazobactam is a β-lactam/β-lactamase inhibitor combination widely employed in first-line therapy, particularly for nosocomial infections (Grant et al., 2002; Fonseca et al., 2004; Lodise et al., 2007). Based on some studies, treatment with subinhibitory concentrations of antibiotics may be effective on bacterial virulence factors, such as adherence, motility and biofilm formation (Wolter & McCormack, 1998; Wilson et al., 2002; Fonseca et al., 2004). The emergence of multidrug-resistant pathogens including cephalosporins and fluoroquinolones has led to the use of Piperacillin/Tazobactam. On the other hand, Piperacillin/Tazobactam is considered a safe antimicrobial agent and has fewer side effects than penicillin derivatives.

Therefore, for the treatment of infections caused by extended-spectrum β-lactamase (ESBL) strains it has been proposed as a substitute to carbapenems (Hall et al., 2019). Despite the good performance of these antibiotics in controlling pseudomonas infections physicians face increasing bacterial resistance, which results in treatment failure. Accordingly, developing new antibacterial agents is necessary to ensure safety and antibacterial activity that does not cause bacterial resistance. In order to improve the performance of antibacterial agents, a variety of antibacterial agents such as peptides, cationic polymers, carbon nanomaterials, and polymeric nanoparticles have been widely studied (Taylor & Webster, 2011; Liu et al., 2019). Therefore, the main objective of this study was to investigate the antibacterial properties of Piperacillin/Tazobactam loaded micelles against planktonic *Pseudomonas aeruginosa*, the ability to eradication biofilm formation and inhibit bacterial motility.

## Materials and methods

### Synthesis of Polylactide-Glycolic acid-polyethylene glycol (PLGA- PEG) copolymer

To prepare PLGA-PEG copolymer, pure powder of lactide (3,6-dimethyl-1, 4-dioxane-2, 5-dione) and glycolide (Sigma Aldrich, American) with a molar ratio of 3 to 1 was added to a three-necked flask. Then 0.91 gr of PEG2000 (Sigma-Aldrich, American) were mixed into the flask with a weight ratio of PLGA to PEG of 2/1 and placed on the heater at 130 °C under argon flow. After melting the materials, the nitrogen gas, 2 drops of Tin octanoate (Sigma Aldrich, American) were added and the reaction continued under argon flow and 130 °C for 24 hours. After 24 hours, the obtained copolymer was dissolved in chloroform (Merck, Germany) and precipitates in cool diethyl ether (Acrose-Belgium) and filtered to separate the pure copolymer. The residual of the solvent was evaporated in a vacuum oven for 24 h.

### Preparation of piperacillin/Tazobactam-Loaded PLGA-PEG micelles

PLGA-PEG polymer was dissolved in Dimethyl sulfoxide (Merck, Germany) solution. Then Piperacillin/Tazobactam powder was added to this solution and stirred for 24 h under dark condition. The weight ratio of PLGA-PEG to Piperacillin/Tazobactam was 10 to 1. Then the polymer-drug solution was added dropwise to polyvinyl alcohol solution (1 wt %) during homogenizing with a probe-type sonicator. The obtained micelles were separated from unloaded drug solution by centrifugation using AmiconV Ultra-15 (molecular weight cutoff of 100 kDa, Millipore, Germany) tube. The formulation was added to the upper chamber of the AmiconV R tube and then the tube was centrifuged at 4000 rpm for 30 minutes. The clear solution at the bottom of AmiconV R tube was used for Piperacillin/Tazobactam determination using a validated spectroscopy method at 240 nm. The encapsulation efficiency (EE) and drug loading capacity (DL) were calculated using the following formula respectively:

EE%=Weight of drug in micelles/Weight of initial drug×100

DL%=Weight of drug in micelles/Weight of micelles containing drug×100

### Characterization of piperacillin/tazobactam loaded micelles

To ensure the accuracy of polymer synthesis, the Proton Nuclear Magnetic Resonance (H-NMR), Carbon Nuclear Magnetic Resonance (CNMR) and frontier transformed infrared resonance (FTIR) Spectroscopy were performed. After micelle preparation, the size, Zeta Potential, Polydispersity Index and surface morphology of the prepared nanoparticles was studied with Dynamic Light Scattering (DLS zeta) (Zeta sizer Nano Zs, Malvern Instruments Ltd, UK), Transmission Electron Microscope (TEM) and Scanning Electron Microscope (SEM). The FTIR analysis and zeta-potential study were performed to confirm Piperacillin/Tazobactam encapsulation into the PLGA-PEG micelles.

### Bacterial isolates and susceptibility testing

In this study, 320 samples of patients including wound, blood, urine and body fluids samples were cultured on Blood agar, Mac Conkey agar and Cetrimide Agar (High media, India). By routine microbiologic methods, 80 strains of *P. aeruginosa* were identified. These isolates were stored in Tryptic Soy Broth (Merck, Germany) contain glycerol at 70 °C for further experiments. Antibiotic susceptibility test was performed using the disc diffusion agar method according to the CLSI recommendation (Cockerill & Patel, 2015). The susceptibility pattern of these isolates was studied against Piperacillin/Tazobactam (100/10 µg), Colistin (10 µg), Ciprofloxacin (5 µg), Norfloxacin (10 µg), Kanamycin (30 µg), Gentamicin (10 µg), Meropenem (10 µg), Imipenem (10 µg), Doripenem (10 µg), Cefepime (30 µg), Ceftazidime (30 µg), Ceftriaxone (30 µg) and Aztreonam (30 µg). Twenty strains that resistant to most antibiotics were included in further testing. To quality control of the antibiotic disks (Bio Maxima, Poland), according to the CLSI protocol, the standard strains of *S. aureus* ATCC 1523 and *E. coli* ATCC 25922 and *P. aeruginosa* ATCC 27853 were used.

### Minimum inhibitory concentration (MIC) of free and piperacillin/tazobactam micelle

The determination of MIC was carried out using a microbroth dilution method (Cockerill & Patel, 2015). Serial concentrations of antibiotics were prepared in CAMHB (cation-adjusted Mueller-Hinton broth) culture media (High media, India). As the concentration of the antibiotic was adjusted range from512 to 0.01 µg/ml. Then Suspensions of 24-hour old cultures were prepared in sterile normal saline to a density equal to No. 0.5 McFarland turbidity standard according to the CLSI recommendation (Cockerill & Patel, 2015) and inoculated into the culture medium contains antibiotic. After incubation at 37 °C, inhibition of growth bacterial isolates was investigated for three consecutive days. MIC was defined as the lowest concentration that inhibited detectable growth. This experiment was conducted separately for both free and Piperacillin/Tazobactam micelle.

### Effect of free or piperacillin/tazobactam micelle against minimal biofilm eradication concentration (MBEC)

Before all, biofilm production was evaluated for all isolates and biofilm-producing isolates were identified. In this study biofilm formation was examined in microtiter plates and interpretation of the results was based on the previous study (Stepanovic et al., 2007). Briefly, the 180 µl CAMHB media containing 0.5% glucose was poured into each well. The bacterial suspension equal to 0.5 Mc Farland was added to the wells. After overnight incubation, the contents of the wells removed and washed three times with sterile phosphate buffer. Then 150 µl of methanol was added to the wells and was incubated at room temperature for 20 min. staining was performed by adding 150 μl of crystal violet for 15 min at room temperature. The plates were then washed and completely dried. Finally, 150 μl of 33% acetic acid was added to the wells and was incubated at room temperature for 30 min. The optical density (OD) of each well is measured at 570 nm by a microtiter plate reader. We used the well-containing media only as a negative control. According to the previous study, the cut off optical density for biofilm formation was considered as follow;

No Biofilm formation (0), weakly (+), moderate (++) and strong formation (+++) were recorded. In order to optimize results and provide reliable analysis of data, the experiment was repeated in three wells per strain.

All these steps were performed in the presence of free drug forms and piperacillin/tazobactam micelle. As the serial dilutions of the antibiotic both forms separately were prepared in wells containing culture media and then the next steps were performed. Wells without antibiotic and wells containing media only were considered as positive and negative controls, respectively.

### Effect of free or piperacillin/tazobactam micelle against bacterial motility

A motility test was performed for all isolates. Cultivation of bacterial isolates was done on nutrient-containing decreased agar concentration. Then bacterial motility was investigated at the surface and depth of culture media. As 1% agar for twitching and 0.5% agar for swarming were used in this study. Ten μl of 0.5 McFarland suspension overnight cultures bacteria was inoculated onto a well created on the surface of the plate and was incubated at 37 °C. After 24 h, swimming and swarming diameters were investigated.

In the same way, the inhibition of bacterial motility was investigated in the presence of different concentrations of the two forms of antibiotic. Assays were performed on each of the total isolates grown overnight with 0.5 MIC of piperacillin/tazobactam. In this study control, positive plates contained no antibiotics.

## Results and discussion

### PLGA-PEG copolymer characterization

**Figure 1.**
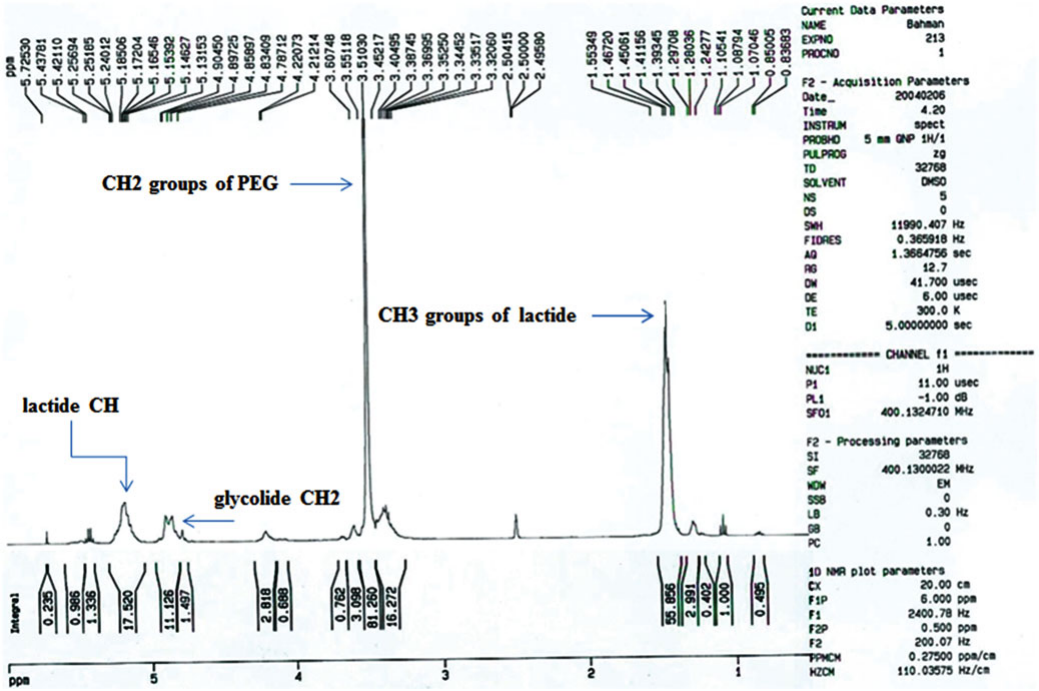
Proton Nuclear Magnetic Resonance spectra of the Polylactide-Glycolic acid-polyethylene glycol micelle.

**Figure 2.**
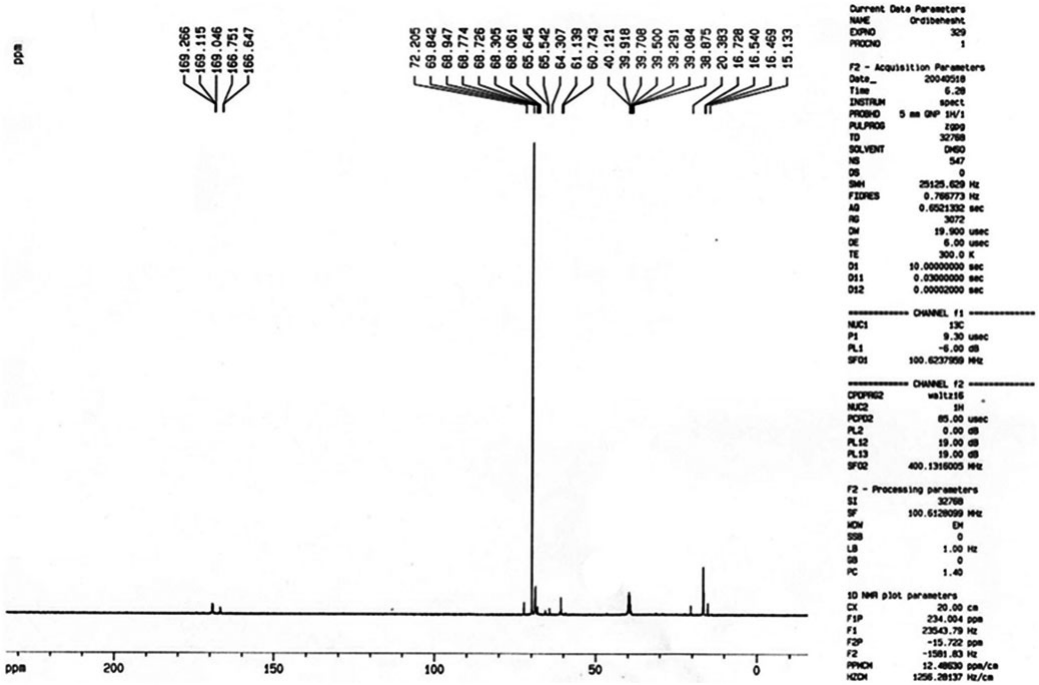
Carbon Nuclear Magnetic Resonance spectra of the Polylactide-Glycolic acid-polyethylene glycol copolymer micelle.

The chemical structure of the PLGA-PEG copolymers was studied with ^1 ^H-NMR and ^13 ^C-NMR by integrating the signals related to each monomer (Figures 1 and 2). As an example, Figure 1 represented the ^1 ^H-NMR spectrum of the PLGA-PEG. The multiples at 5.2 and 4.9 ppm correspond to the lactide CH and the glycolide CH2, respectively and the peak at 1.467 relates to lactide CH3. Also, the large peak at 3.51 ppm corresponds to the methylene groups of PEG. Figure 2 shows the ^13 ^C-NMR spectrum of PLGA-PEG. ^13 ^C-NMR analysis of PLGA-PEG showed the peak at 171.6 and 169.1 ppm corresponds to carboxylic and carbonyl bonds. The peaks at 60.7 and 72 ppm were related to CH bonds in lactic acid and CH_2_ in glycolic acid, and the peak appeared at 16.7 ppm corresponding to methylene groups of the d, l-lactic acid repeated units.

**Figure 3.**
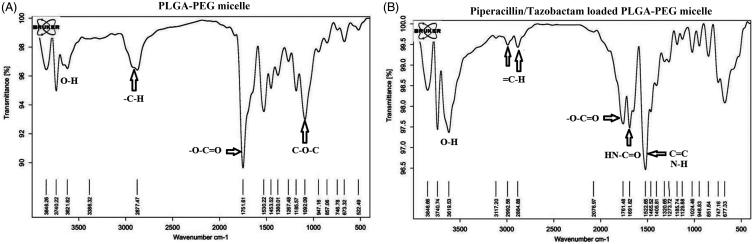
Frontier Transformed Infrared Resonance spectra of the Polylactide-Glycolic acid-polyethylene glycol (PLGA-PEG) micelle (A) and Piperacillin/Tazobactam loaded PLGA-PEG micelle (B).

The Fourier transform infrared (FT-IR) spectra of the PLGA-PEG are shown in Figure 3. Prominent peak at 1751 cm^−1^ corresponded to C = O bonds of lactide and glycolide moiety of micelle and etheric bonds (C-O-C) appear at 1092 cm^−1^. The hydroxyl groups of the polymer were observed at 3600 cm^−1^. The bond at 2877 cm^−1^ clearly indicates the aliphatic C-H groups of the polymer backbone. After tazobactam and piperacillin loading on PLGA-PEG, the appearance of new peaks at 2992.56 and 3117.20 cm^−1^ corresponds to aliphatic and aromatic protons of = C-H presented in tazobactam and piperacillin, respectively. The presence of a new peak at 1691 cm^−1^ corresponds to amide (HN-C = O) and carboxylic (O-C = O) groups and the peak at 1405–1522 cm^−1^ relates to C = C at piperacillin and tazobactam. Therefore, FTIR spectra proved successful encapsulation of piperacillin and tazobactam on PLGA-PEG micelles.

### Size and morphology study of PLGA-PEG micelle

**Figure 4.**
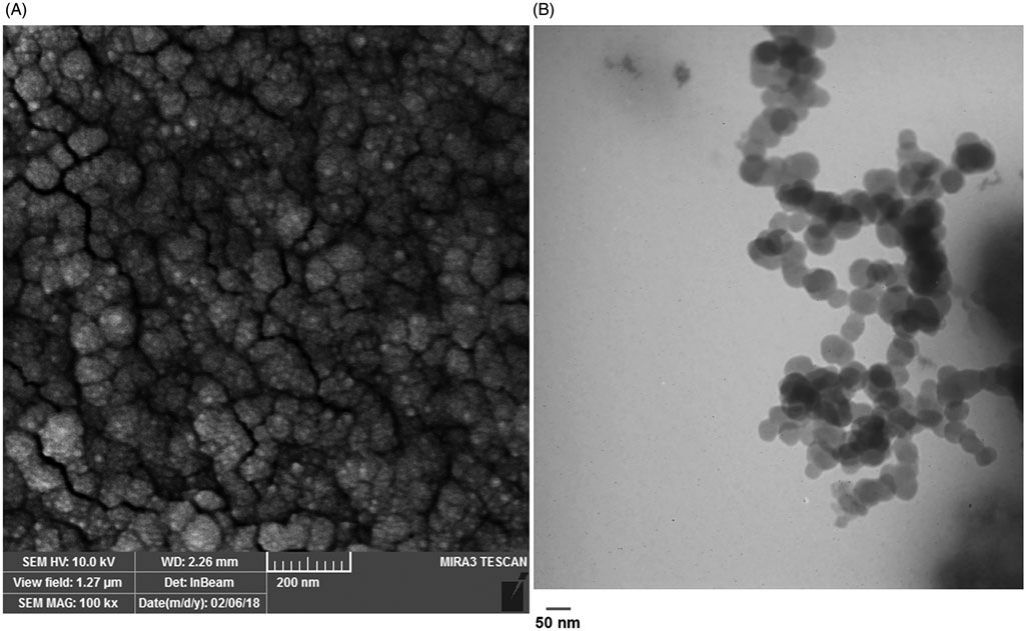
Size and morphology determination of the PLGA-PEG micelle assessed by Scanning Electron Microscope (A) and Transmission electron microscope (B).

SEM and TEM studies showed the PLGA-PEG micelle, semi-spherical morphology with a mean diameter of below30 nm obtained by image J software (Figure 4).

In our research, DLS studies indicated that micelles were prepared with a homogeneous size of around 22 nm (Figure S1).

**Figure 5.**
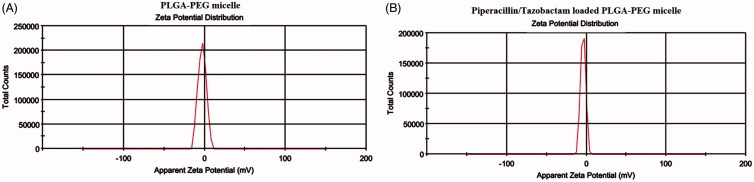
Surface charge of PLGA-PEG micelle (A) and piperacillin/tazobactam loaded PLGA-PEG micelle (B) determined by Zeta sizer.

The Zeta potential results indicated that the surface charge of PLGA-PEG micelle was −2.98 mV, while after the encapsulation of Piperacillin/Tazobactam, the surface charge decreased to −4.13 mV (Figure 5).

Fabrication of polymeric micelle containing piperacillin/tazobactam was further verified by UV − vis absorption spectra. The presence of absorption peak at 240 nm was related to the presence of piperacillin/tazobactam in nanomicelle formulation (Figure S2). Also, encapsulation efficiency (EE) and drug loading (DL) capacity were calculated after the procedure. In our study, encapsulation efficiency (EE) and drug loading (DL) capacity were 75% and 15%, respectively.

### Antibacterial studies

In this study, 320 specimens including wound, blood, urine and body fluids were cultured and 80 strains of *P.aeruginosa* were identified (Figure S3). Today, increasing the prevalence of multidrug-resistant *P. aeruginosa* strains is a major problem. In developing countries such as Iran, due to multiple factors, multidrug resistance is high. This issue constantly limits the treatment options for *P. aeruginosa* infections (Khosravi et al., 2019). Antibiotic susceptibility test was performed by disc diffusion agar method according to CLSI guideline (Patel, 2017). The results of the antibiotic susceptibility test showed that the highest resistance was found against ceftriaxone, Kanamycin, and Ceftazidime (Table 1). Additionally, the lowest resistance was found against Colistin and Doripenem. In the previous study in Iran (Forozsh et al., 2012), all isolates have been reported to be susceptible to Imipenem, Ticarcillin, ciprofloxacin, and piperacillin, while the lowest susceptibility was reported to Ceftazidime and Tobramycin. Despite different results that are probably due to geographical differences, we found resistance to Cefepime, Imipenem, Meropenem, and Ceftazidime was 100%, 81%, 41%, and 23%, respectively, which is confirmed by other studies (Khosravi & Mihani, 2008).

**Table 1.** Pattern of resistance and sensitivity of *P. aeruginosa* isolates to antibiotics tested.

Isolates	Antibiotic discs												
	COL (%)	IMP (%)	DOR (%)	MEM (%)	NOR (%)	FEP (%)	PIT (%)	CP (%)	CN (%)	ATM (%)	CAZ (%)	K (%)	CRO (%)
Resistance	23.2	36.7	39	39.8	43	49.8	51.5	55	56.6	56.6	59.7	63.3	70
Non-Susceptible	0	15	9	8.2	6.6	10	8.5	1.7	10	5	6.6	6.7	3.3
Susceptible	56.8	28.3	32	32	29.9	20.2	20	23.3	13.4	18.4	13.7	10	6.7

COL: Colistin; IMP: Imipenem; DOR: Doripenem; MEM: Meropenem; NOR: Norfloxacin; FEP: Cefepime; PIT: piperacillin/tazobactam; CP: Ciprofloxacin; CN: Gentamicin; ATM: Aztreonam; CAZ: Ceftazidime; K: Kanamycin; CRO: Ceftriaxone.

**Figure 6.**
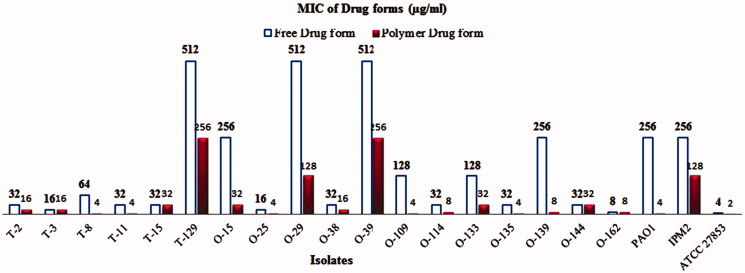
Minimum Inhibitory Concentration of Piperacillin/Tazobactam loaded PLGA-PEG micelle and free Piperacillin/Tazobactam in some strains of *P. aeruginosa*.

Then minimum inhibitory concentration of two forms of the drug was determined. In this study, the results of MIC showed that there was a significant difference in the minimum inhibitory concentration of these forms in some strains of *P. aeruginosa* (Figure 6). Notably, much lower MIC values were observed for Piperacillin/Tazobactam-loaded PLGA-PEG micelle than for free ones, suggesting more potent antibacterial activities of Piperacillin/Tazobactam-loaded PLGA-PEG micelle. The effect of these materials on bacterial strains was evaluated at 24, 48, and 72-hours intervals. Interestingly, increased incubation time had no effect on the results. Thus, the antibacterial activity of Piperacillin/Tazobactam was improved when loaded with PLGA-PEG micelle against *p. aeruginosa* isolates. Similar to our results, Takahashi et al. (2017) showed that the viability of bacterial cells decreased after treatment with Clarithromycin encapsulated + chitosan modified polymeric nanoparticles and Clarithromycin encapsulated + chitosan modified Sol micelle. They concluded that CAM-encapsulated and chitosan modified Sol micelle had a higher antibacterial activity. In two separate studies, we showed that Azithromycin-loaded NPs were more effective than the free drug against *S. typhi* strain and Clarithromycin-loaded PLGA NPs were more effective than those of untreated CLR against all tested isolates (Mohammadi et al., 2010; Valizadeh et al., 2012). Fan Huang et al. (2017) revealed that silver-decorated micelles had excellent antibacterial property against *P. aeruginosa* and *S. aureus* strains. They emphasized that when the silver-decorated polymeric micelles were combined with curcumin, the antibacterial property noticeably increased. Finally, it can be said that a variety of nanoparticles can improve and increase the antimicrobial effect of the compounds. Also, Farhangi et al. (2018) in Iran showed that encapsulated ciprofloxacin in chitosan nano micelles had a greater effect on *P. aeruginosa* and *K. pneumoniae* species compared to the free drug form. Similar to our study, synthesized nanoparticles have considerably reduced the MIC in comparison with the free drug form.

Motility plays a key role in bacterial pathogenicity because it is essential for bacterial colonization and the beginning of an infection. The motility of bacteria and virulence are closely related. Therefore, it is possible to use bacterial mobility as an antibacterial therapeutic target for the treatment or prevention of infectious disease (Josenhans & Suerbaum, 2002).

In our study, only in eight isolates motility inhibition including twitching and swarming was observed in the presence of similar concentrations of P/T loaded PLGA-PEG micelle and free drug forms. However, in other isolates, motility inhibition occurred in low concentrations of P/T loaded PLGA-PEG micelle than free drug form. As in the presence of this drug form, the concentration of motility inhibition decreased from 2 to 8 times (Figure S4).

de Andrade et al. (2016) revealed that the presence of 0.5 × MIC of Piperacillin/Tazobactam can inhibit bacterial motility. Fonseca et al. (2004) evaluated the ability to swim using flagella and to twitch of *P. aeruginosa* strains in the presence of sub-inhibitory concentrations of Piperacillin/Tazobactam. Their results indicate that sub-MIC P/T interfere with the ability to move *P. aeruginosa*. The results obtained from our study also confirmed by the results of current studies.

The biofilm formation in bacteria is a defense mechanism that protects them against the penetration of antimicrobial agents, especially antibiotics. This feature plays an important role in the clinical outcome of the *P. aeruginosa* infections. Researchers emphasize that in order to overcome *P. aeruginosa* infections, we should be able to eradicate biofilm formation (Hamilos, 2019; Suresh et al., 2019). First of all, we evaluated the biofilm formation in all the isolates. Evaluation of biofilm formation showed that all tested strains, except one isolate, were positive at a strong level. Then, the minimal biofilm eradication concentration in these strains was investigated in the presence of P/T-loaded PLGA-PEG micelle and free drug form. We showed that biofilm was eradicated in 7 strains in the presence of P/T-loaded PLGA-PEG micelle ranging from 512 to 0.12 μg/ml. However, the biofilm eradication in these isolates was seen in the presence of free drug form ranging from 512 to 1 μg/ml. Overall, our results showed that the micelle form can eradicate the biofilm formation at low concentrations in comparison with free drug form. Details are shown in Table 2.

**Table 2.** Minimum biofilm eradication concentration of P/T loaded PLGA-PEG micelle and free drug form on *P. aeruginosa*.

Isolates		Minimum Biofilm Eradication Concentration Assay (MBEC µg/ml)														
		512	256	128	64	32	16	8	4	2	1	0.5	0.25	0.12	0.06	0.03
T-2	MDF	0	0	0	0	0	0	0	0	0	0	0	0	0	w	w
	FDF	0	0	0	0	0	0	0	0	0	0	w	w	W	w	m
T-3	MDF	0	0	0	0	0	0	0	0	0	0	0	0	0	0	w
	FDF	0	0	0	0	0	0	0	0	0	0	w	m	M	m	s
T-8	MDF	0	0	0	0	0	0	0	0	0	0	0	0	W	w	m
	FDF	0	0	0	0	0	0	0	0	0	0	w	m	S	s	s
T-11	MDF	0	0	0	0	0	0	0	0	0	0	0	w	W	w	w
	FDF	0	0	0	0	0	0	0	0	0	0	m	m	M	w	w
T-15	MDF	0	0	0	0	0	0	0	0	0	0	0	0	0	w	w
	FDF	0	0	0	0	0	0	0	0	0	0	m	m	M	s	s
T-129	MDF	0	0	0	0	0	0	0	0	0	0	w	W	W	m	m
	FDF	0	0	0	0	0	0	0	0	0	0	w	w	M	m	s
O-15	MDF	0	0	0	0	0	0	0	0	0	0	w	s	S	s	s
	FDF	0	0	0	0	0	0	0	0	0	0	w	s	S	s	s
O-25	MDF	0	0	0	0	0	0	0	0	0	0	0	0	0	0	w
	FDF	0	0	0	0	0	0	0	0	0	0	w	m	m	s	s
O-29	MDF	0	0	0	0	0	0	0	0	0	0	s	s	s	s	s
	FDF	0	0	0	0	0	0	0	0	0	w	w	w	m	m	s
O-38	MDF	0	0	0	0	0	0	0	0	0	0	0	w	w	w	s
	FDF	0	0	0	0	0	0	0	0	0	0	w	w	m	m	s
O-39	MDF	0	0	0	0	0	0	0	0	0	0	w	s	s	s	s
	FDF	0	0	0	0	0	0	0	0	0	m	s	s	s	s	s
O-109	MDF	0	0	0	0	0	0	0	0	0	0	w	w	m	s	s
	FDF	0	0	0	0	0	0	0	0	0	w	w	m	m	m	s
O-114	MDF	0	0	0	0	0	0	0	0	0	0	0	m	m	m	s
	FDF	0	0	0	0	0	0	0	0	0	0	m	m	s	s	s
O-133	MDF	0	0	0	0	0	0	0	0	0	0	w	m	m	s	s
	FDF	0	0	0	0	0	0	0	0	0	w	m	s	s	s	s
O-135	MDF	0	0	0	0	0	0	0	0	0	0	0	0	w	w	w
	FDF	0	0	0	0	0	0	0	0	0	0	0	0	w	m	s
O-144	MDF	0	0	0	0	0	0	0	0	0	0	0	w	s	s	s
	FDF	0	0	0	0	0	0	0	0	0	0	s	s	s	s	s
O-162	MDF	0	0	0	0	0	0	0	0	0	0	0	w	w	s	s
	FDF	0	0	0	0	0	0	0	0	0	0	w	s	s	s	s
PAO1	MDF	0	0	0	0	0	0	0	0	0	0	0	0	0	w	w
	FDF	0	0	0	0	0	0	0	0	0	0	w	w	w	s	s
IPM2	MDF	0	0	0	0	0	0	0	0	0	0	0	0	0	0	w
	FDF	0	0	0	0	0	0	0	0	0	0	0	0	0	w	w
ATCC 27853	MDF	0	0	0	0	0	0	0	0	0	0	0	0	0	0	w
	FDF	0	0	0	0	0	0	0	0	0	0	w	w	s	s	s

Micelle drug form: MDF; FDF: Free drug form; 0 (no biofilm formation), W (weakly or +), m (moderate or ++), s (strong or +++) biofilm formation.

Bora Onat et al. (2016) demonstrated that Triclosan loaded-micelles are effective against *S. aureus* and *E. coli* biofilm. Also, Su et al. (2018) revealed that Triclosan loaded into PEG-g-PU micelles had more potent anti-biofilm activities. Thus, they claimed that PEG-g-PU micelles could be used as hydrophobic antibiotic carriers to treat microbial infections and biofilm eradication. Liu et al. (2016) were indicated that micellar nanocarriers fully penetrate and accumulate in a biofilm layer of *S. aureus*. Also, Liu et al. (2018) that PEG-PAE micelles with conjugated antimicrobials can uniquely penetrate biofilms. The results of their study demonstrated that PEG-PAE-T micelles due to the excellent penetration properties of those into biofilms and the extremely localized release of Triclosan inside the biofilm, cause bacterial killing and biofilm eradication. All these studies confirm the results of our research. As we found, the micelle nanoparticles have a more impact on bacterial killing and biofilm eradication.

## Conclusion

In conclusion, we developed a micellar nanocarrier based formulation that increases the effectiveness of the Piperacillin/Tazobactam. Based on our data, Piperacillin/Tazobactam loaded PLGA-PEG micelle is more effective against resistant *P. aeruginosa* strains compared to free drug form. We indicated that the PLGA-PEG micelles can be good carriers to deliver antibiotics into bacterial biofilms and eradicating biofilm formation. In general, our observations suggest that the above formulation can be an attractive strategy for controlling resistant *P. aeruginosa* infections.

## Supplementary Material

Supplemental Material
