# Supplementary material for: Synthesis and evaluation of polymeric micelle containing piperacillin/tazobactam for enhanced antibacterial activity
Source: Drug Deliv. 2019 Dec 4;26(1):1292–9. doi: 10.1080/10717544.2019.1693708 (PMC6896493; doi:10.1080/10717544.2019.1693708)
Supplement: Supplemental Material [file IDRD_A_1693708_SM8946.docx]

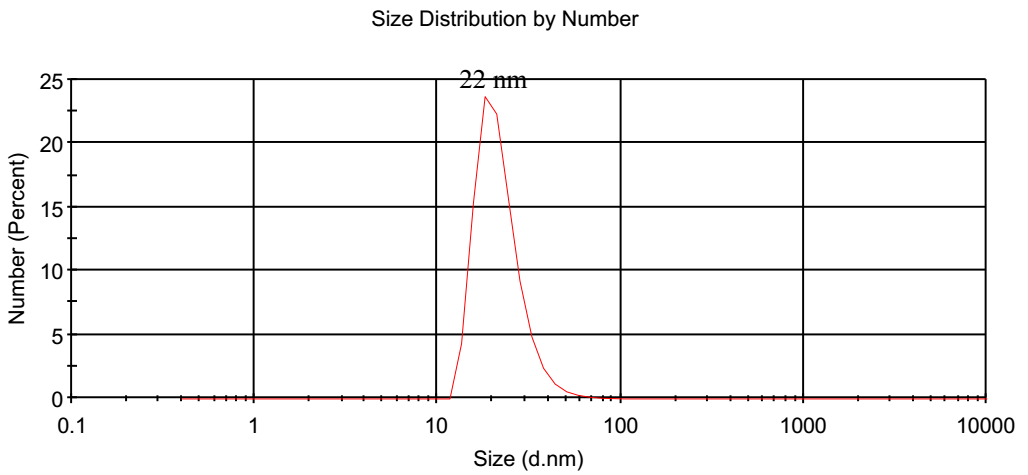


Figure S_1_: Dynamic light scattering results of PLGA-PEG micelle


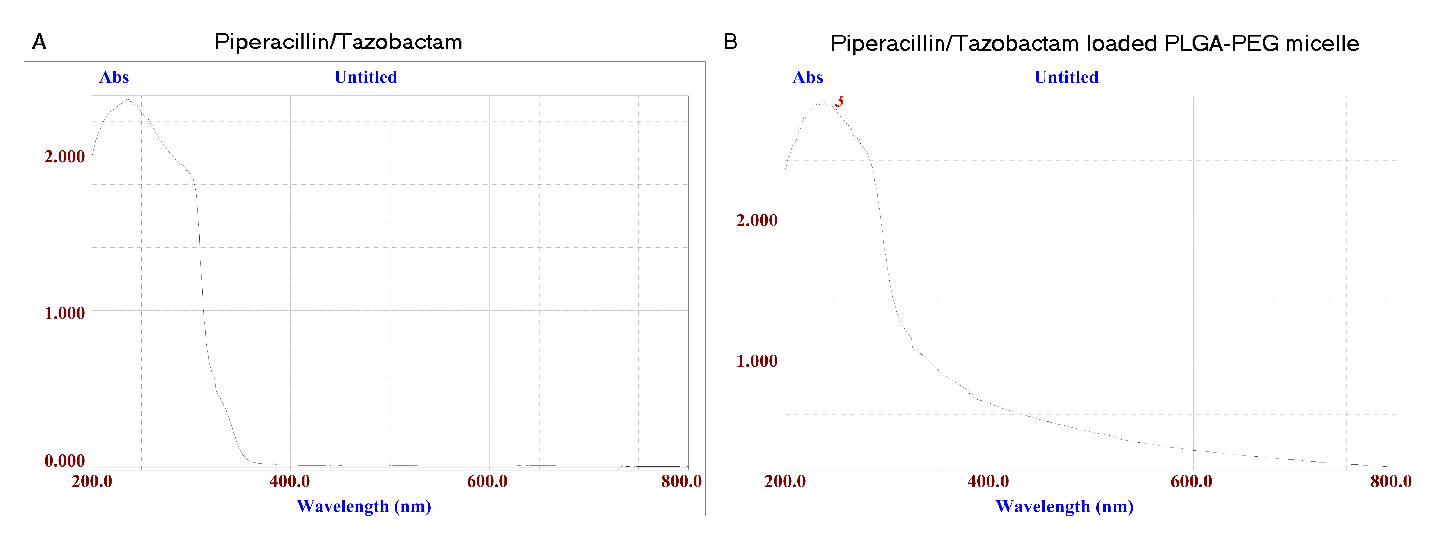


Figure S2: Uv-Vis spectra of A) Piperacillin/Tazibactam and B) Piperacillin/Tazibactam loaded PLGA-PEG micelle


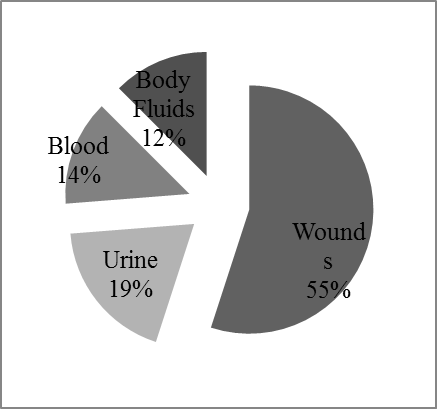


Figure S_3_: Frequency of *p. aeruginosa* isolated from various tissues of the body

Figure S_4_: Motility inhibition test of free and polymer drug form against on *p. aeruginosa* isolates
